# Communication as a Strategy to Promote Sports and Health Activities Designed for Adolescents

**DOI:** 10.3390/ijerph17134861

**Published:** 2020-07-06

**Authors:** Antonio Fernández-Martínez, Raquel Pérez-Ordás, Román Nuviala, Mónica Aznar, Ana María Porcel-Gálvez, Alberto Nuviala

**Affiliations:** 1 Department of Sports and Computer Science, Pablo de Olavide University, Crta. de Utrera, Km1, 41013 Seville, Spain; anuvnuv@upo.es; 2 Faculty of Human Sciences and Education, University of Zaragoza, Valentín Carderera, 4, 22003 Huesca, Spain; rpordas@unizar.es (R.P.-O.); moaznar@unizar.es (M.A.); 3 Faculty of Education Sciences, University of Cádiz, Avda. República Saharaui, s/n. Campus de Puerto Real, 11519 Puerto Real, Cádiz, Spain; roman.nuviala@gm.uca.es; 4 Department of Nursing, University of Seville, Calle Avenzoar 6, 41089 Seville, Spain; aporcel@us.es

**Keywords:** adolescents, sports and health services, communication, satisfaction, future intentions

## Abstract

Physical activity reduces the risk of developing noncommunicable diseases and improves quality of life, providing health benefits for present and future generations. This is especially relevant for adolescents. Educational institutions are promoters of healthy habits through the organisation of different activities such as extracurricular sports programmes. These activities increase the rates of sports practice among adolescents. The literature shows that the perceived quality of sports and health services is an antecedent of users’ behavioural intentions. The aim of this paper is to find out whether communication from educational/sports organisations influences adolescents’ intentions to continue engaging in physical activity. A total of 1080 students participated, with a mean age of 13.76 ± 1.39 years, 34.1% of whom were girls. Tests were conducted to verify the validity and reliability of the model that relates communication with value, satisfaction, and future intentions. Tests were conducted to verify the validity (average variance extracted was between 0.754 and 0.583) and reliability (composite reliability was between 0.925 and 0.813) of the model that relates communication with value, satisfaction, and future intentions. Confirmatory analyses and factor invariance tests were performed. The results revealed that communication is an antecedent of value, satisfaction, and future intentions. In conclusion, communication is a good strategy to consolidate sporting habits in both male and female adolescents.

## 1. Introduction

Numerous studies show the importance of physical activity in improving public health [1]. Physical inactivity is considered to be one of the main factors behind morbidity and mortality worldwide [2]. A growing body of research shows that regular physical activity reduces the risk of developing noncommunicable diseases [3] and improves the health of the population. This is especially important at early ages, when more than 80% of adolescents between 11 and 17 years of age do not engage in sufficient physical activity [4]. Scientific evidence shows the fundamental health benefits of physical exercise for present and future generations, with physical activity interventions being associated with improved health indicators [5].

This is compounded by socioenvironmental factors that do not encourage the adoption and maintenance of an active lifestyle, such as pollution, traffic, lack of green areas or sports facilities, and difficulty in accessing sustainable, safe, and active mobility [6,7]. Added to this are the changes in the way of playing or spending leisure time, where sedentary activities linked to technological games prevail [8,9].

In this context, educational institutions can play a fundamental role as promoters of healthy habits by educating and promoting physical activity [10,11]. The organisational structures of educational institutions, their relationship with the community, and their facilities for sports practice are suitable elements for the promotion of physical activity inside and outside of school [12,13,14].

The extracurricular sports activity programmes offered by educational institutions have been shown to improve physical practice rates in adolescents [15]. However, despite these good results, there is still a high dropout rate in this age group [16]. This is why it is necessary to increase loyalty to the range of physical activities offered by educational institutions, as this will allow a greater number of students to acquire and maintain healthy habits [17]. This acquisition of habits cannot be understood exclusively as the repetitive practice of activities; it must also include the acquisition of different health-related skills by establishing bonds of trust or loyalty with these programmes and organisations [18,19]. Loyalty to these activities involves the development of behavioural aspects (frequency of practice), different attitudes (such as recommending behaviours), and even cognitive achievements (knowledge of habits) [20].

Loyalty to sports activities, programmes, and organisations has quality, value, and satisfaction as antecedents [21,22,23]. Quality may be defined as the users’ impression or judgement of the excellence, superiority or inferiority of a product, and an organisation and/or its services [24,25], and its measurement must be adapted to the context under examination [26,27,28]. Value is defined as the consumers’ overall assessment of the utility of a product, based on their perception of what is received and what is given [29]. Value is, therefore, the result of comparing the sacrifices and benefits that the service brings to customers [30]. Finally, satisfaction may be understood as the general assessment of the service in comparison with user expectations [31,32], and is the result of both cognitive and affective perceptions [33].

Thus, sports services with low ratings in relation to quality, value, and satisfaction have high dropout rates, results that can be observed in both sports and health services targeting adults and/or adolescents [34,35]. Studies can be found in the literature on the relationships between these constructs [21,22,23,36]. However, only Pérez-Ordás et al. [17] have conducted a study with adolescents in which they examined whether quality, value, and satisfaction were direct antecedents to future intentions. In this study, they analysed the direct relationships between the dimensions that make up quality, value, and satisfaction, and the dimensions that make up the construct of future intentions in this age group. However, there are no studies analysing whether quality and its component dimensions are directly related to value and satisfaction, or indirectly related to future intentions through these constructs in this particular age group.

Quality is the primary antecedent in sports service measurement models [36]. Nuviala et al. [37] designed a tool to assess the quality of a large group of sports organisations. Quality was measured through six factors, one of which was the organisation’s communication, which included items relating to information campaigns of the sports and health service targeting external users. Communication within organisations, including educational organisations, is a determining factor in the success of the process of service provision [38]. Communication is a key element in the development of motivation and the participation of the agents that are involved in achieving the objectives, dissemination, and understanding of the educational process [39], being especially relevant for adolescents. Increased levels of communication between users and service providers build feelings of trust among users [40], which in turn generates greater user loyalty to the organisation.

In addition, quality is an antecedent of value [21,35,37], which means that value becomes a strategy on which the success of sport and health service organisations depends [41]. Since no studies on the relationship between communication and the constructs of value, satisfaction, and the dimensions of future intentions have been found in the literature, nor has this relationship been studied in this specific population, the following hypothesis can be formulated: communication is an antecedent of perceived value in school-based sports services (H1).

Several articles state that quality is an antecedent to satisfaction [42,43]. Organisations wishing to increase the levels of satisfaction of their users adopt the strategy of increasing quality levels through the direct and significant relationship between both constructs [36]. Any action taken by the managers of sports and health services will have an impact on satisfaction. On the basis of this evidence, the following hypothesis can be established: communication, as a dimension of quality, is an antecedent to satisfaction in school-based sports services (H2).

In the literature on sports and health service management, studies have shown that service quality influences users’ future intentions [43,44]. Future intentions may be understood as users’ favourable attitudes towards the sports and health service when it comes to recommending the centre and its services and showing positive repurchase behaviours [45]. The concept of future intentions is a broad concept that encompasses the concept of loyalty, lies within an attitudinal view of users, and is often used in the study of user and consumer behaviours [46].

Future intentions are composed of different dimensions [47,48]. One of the most widely accepted measurement tools in the literature is that proposed by Zeithaml et al. [49], which was adapted for use in sports services by Nuviala et al. [50], resulting in three dimensions (loyalty, adaptation to price, and responsiveness), components of the future intentions of the users of this type of service. Thus, knowing that quality is an antecedent to the future intentions of sports service users, the following hypotheses can be formulated: in school-based sports services, communication is an antecedent to loyalty (H3); communication is an antecedent to adaptation to price (H4); communication is an antecedent to responsiveness (H5).

Therefore, the objective of this study is to determine if communication in educational/sports organisations affects the intentions of adolescents to continue engaging in physical activity.

## 2. Materials and Methods

### 2.1. Subjects

A total of 1080 students in compulsory secondary education from a city in northern Spain participated in the study. All educational institutions offering extracurricular physical and sports activities, 62 in total, were asked to participate in this research. Twenty-seven educational institutions agreed to participate. All of the schoolchildren from these educational institutions who carried out physical sports activities during out-of-school hours were selected to participate in the study, with the exclusion criterion being participating in extracurricular sports activities for less than one hour a week. The students had a mean age of 13.76 ± 1.39 years, 34.1% of them were female, 90.9% of them were engaged in competitive activities (official competitions organised by sports federations), and 9.1% were engaged in recreational physical activities. The most frequent activities were football (soccer) for boys and basketball and dance for girls (Table 1).

### 2.2. Instruments

The following instruments were used as a basis to design the instrument for this study (Table 2): the Sports Organisations Perception Scale, version 2 (EPOD2) [37], the Scale of Future Behavioural Intentions in Users of Sports Services [50], and sociodemographic questions (sex, age, and types of sports activities performed). 

Using the EPOD2 scale, the following aspects were assessed in users of sports and health services: communication (3 items, Cronbach’s alpha: 0.891), perceived value (1 item), and satisfaction (4 items, Cronbach’s alpha: 0.906). These items are rated using a Likert scale ranging from 1 to 5.

The second of these instruments, the Scale of Future Behavioural Intentions in Users of Sports Services, with Likert scales ranging from 1 to 7, was used to assess future intentions to continue to engage in physical activity. It has three dimensions: reaction to price (2 items), responsiveness (3 items), and loyalty (5 items). After collecting data from the participants, internal consistency was verified using Cronbach’s alpha (0.812). 

### 2.3. Procedure and Research Design

This study used a cross-sectional design and met the highest safety and ethical standards. It was approved by the ethics commission of the Regional Government of Andalusia, Spain. Subsequently, authorisation was requested from the various sports organisations that were to take part in the data collection process. The legal representatives of the students were informed of the research to be conducted, and their authorisation and subsequent consent were obtained. Data collection, which took approximately 10 min, was carried out in the presence of a previously trained interviewer. The collected data were handled according to the Spanish data protection law in force at the time (Organic Law 15/1999). The principles enshrined in the Declaration of Helsinki (2013, Brazilian revision) [51] were also observed.

### 2.4. Statistical Analysis

Initially, using the software Statistical Package for the Social Sciences (SPSS, IBM, Armonk, NY, USA), version 22.0, descriptive statistics were calculated (means and standard deviations) and correlations between factors and reliability were analysed. The Average Variance Extracted (AVE) and Composite Reliability (CR) were also calculated using an Excel spreadsheet. With the program Analysis of Moment Structure (AMOS, IBM, Armonk, NY, USA), version 22.0, a multigroup analysis was conducted. The aim of this statistical procedure was to check the invariance of the factor structure in both male and female groups of students. The objective of this analysis was to determine whether the model examined, consisting of the dimensions of communication, value, satisfaction, and future intentions, was reproduced in the same way in the group of girls and in the group of boys. The analysis was carried out using the maximum likelihood procedure.

The adjustment of the models was assessed by examining several indices. The following statistics were used: *χ*^2^ (CMIN), degrees of freedom (DF), the ratio between *χ*^2^ and the number of degrees of freedom (CMIN/DF), the Comparative Fix Index (CFI), the Root Mean Square Error of Approximation (RMSEA), the Akaike Information Criterion (AIC), and Expected Cross Validation Index (ECVI). Finally, for both the boys’ and girls’ models, regression coefficients (both standardised and unstandardised) were calculated for the existing relationships of the factors included.

## 3. Results

As shown in the descriptive statistics of Table 3, the mean of all constructs is between the means of the two scales used (3.86 ± 0.92 for the dimensions of communication, value, and satisfaction; 4.61 ± 1.34 for the scales for loyalty, adaptation to price, and responsiveness). Internal consistency was assessed for each research construct. The results of the CR test revealed that all values were greater than 0.6. The calculation of the AVE suggests convergent validity, since the resulting AVE values of the research constructs were close to or above the recommended value of 0.5. The discriminant validity of the data was verified by calculating the correlation matrix between the factors. As can be observed, there was a significant positive correlation between the factors that make up the study, except for the correlations between responsiveness and satisfaction and between responsiveness and value.

The model relating communication, perceived value, satisfaction, and future intentions (loyalty, adaptation to price, and responsiveness) was then tested to check the validity of the factor structure of the data in the two groups of children (males and females). Appropriate values were obtained for both sexes (model 0a for boys and 0b for girls). The analysed models showed good fit indices, as shown in Table 4.

Subsequently, in order to check the factor invariance of the model and compare the populations with each other, the differences in *χ*^2^ divided by the degrees of freedom (CMIN/DF) between the unrestricted model (model 1) and the different restricted models were considered, revealing the existence of differences (Table 3). It may be observed that all the CFI in the models have very similar values, with a difference between them of less than −0.01, which points to the factorial invariance of the model. Similarly, when looking at the AIC and ECVI indices, the differences in adjustments are minimal, which indicates that the different models have very similar values.

Model 3 was selected to compare the two groups. The values obtained were good and similar for each of the indices, with model 3 presenting a better CMIN/DF coefficient (Table 4).

The data in Table 5 show that the communication dimension has a direct and significant relationship with value in both groups, with the standardised value being higher in girls. In addition, communication has a direct impact on the satisfaction dimension, with similar factor loadings in both boys and girls. 

Communication has a significant relationship with loyalty, with similar *β*-values in both groups. Communication has a direct and significant relationship with the future intention of adaptation to price in both boys and girls. Finally, communication exhibits a significant relationship with responsiveness, with standardised values being similar between the two groups. 

## 4. Discussion

In this study, we assessed the association between the communication dimension, included within the construct of the quality of sports and health services, and the perceived value, satisfaction, and future intentions of school children attending sports activities in educational institutions. We also examined whether the communication dimension had different associations according to gender. For this purpose, two research instruments [37,50], used in other studies on other groups of people, were used. As a result, it was necessary to assess their validity and reliability for use in this population group. The quantitative analysis was conducted by examining the mean score of the factors under study. The results revealed that the values were around the midpoint of the scale and the standard deviation was close to one, which, according to Carretero-Dios and Pérez [52], points to the normality of the results. Reliability was checked with Cronbach’s alpha and correct values were obtained. Convergent validity was determined by evaluating the correlations between the factors under study using Pearson’s coefficient. The correlations between many of the constructs were positive, moderate, and significantly related, which demonstrate this type of validity. It should be added that there were no correlations between responsiveness and satisfaction nor between responsiveness and value. This result may be due to them being different constructs. Responsiveness was included in the instrument, which measures the future intentions of the users of these sports and health services, while value and satisfaction are measures of how sports services are assessed. Additional evidence for the validity of the instrument is provided by the CR and AVE values. Acceptable values are >0.6 for the CR and >0.5 for the AVE [53,54]. Consequently, the results obtained indicate the validity of the instrument, as they were higher than the values suggested in the literature.

After verifying the reliability and validity of the instruments used to assess the association of communication with value, satisfaction, and future intentions of school children attending sports and health activities in educational institutions, which are an essential element in the creation of healthy habits in the adolescent population that will probably extend into adulthood [55], the validity of the model was checked by means of a confirmatory factor analysis. The parameters were estimated using the maximum likelihood method, following Thompson’s recommendation [56]. In order to evaluate the suitability of the model being tested, a group of indices was jointly assessed. The following statistics were used: *χ*^2^ (CMIN), degrees of freedom (DF), the ratio between *χ*^2^ and the number of degrees of freedom (CMIN/DF), the Comparative Fix Index (CFI), the Root Mean Square Error of Approximation (RMSEA), the Akaike Information Criterion (AIC), and the Expected Cross Validation Index (ECVI). It is important to note that CFI values above 0.90 and RMSEA values between 0.05 and 0.08 are considered to be acceptable. Regarding the CMIN/DF ratio, a perfect model would have a value of 1.00, and ratios below 2.00 would be considered to be indicators of a very good model fit, while values below 5.00 are considered to be acceptable [57]. Following Byrne’s [58] guidelines, a model with a better fit than another model would show lower AIC and ECVI values. The model studied in the two groups of users, boys and girls, showed correct adjustment rates, as these rates were in accordance with the critical values, i.e., over 0.90 for CFI and below 0.08 for the RMSEA. The CMIN/DF ratio was less than 3 in the two groups. The AIC and ECVI values are very small, suggesting a good fit. All this shows the correct fit in both population groups, thus allowing factor invariance tests to be performed.

The invariance of the factor structure was analysed through multigroup analysis following the recommendations of Abalo et al. [59]. The aim was to check that there were no significant differences between a model without invariance and different models with invariance in some parameters. Significant differences were found between the unrestricted model (model 1) and the rest of the models. However, since CMIN/DF is sensitive to sample size, the criterion established by Cheung and Rensvold [60] for ΔCFI was also used. According to these authors, ΔCFI values lower than or equal to −0.01 indicate that the null hypothesis of invariance cannot be rejected. The ΔCFI values found in this study in the comparison between the unrestricted model and the rest of the models suggest that the factor structure of the model that relates communication, value, satisfaction, and future intentions is invariant.

A significant relationship was thus found between the communication dimension and value, satisfaction, loyalty, adaptation to price, and responsiveness. In addition, minimal differences were found between the group of boys and the group of girls. According to these results, in general, it is safe to say that there is a relationship between quality, specifically in the communication dimension, and the value judgements of the sports services received, which is in line with other results found in the literature [21,35,37]. An association was also found between communication, a dimension included in the construct of quality, and the adolescents’ future intentions in relation to these educational sports and health services, which serve as a tool for adherence to and maintenance of sports practice, with positive effects on the current and future health of adolescents. This result corroborates the positive effect of quality and its dimensions on the future intentions of users of sports and health services [17,21].

The first of the hypotheses, i.e., that communication is an antecedent of perceived value, was corroborated by the results of this research. The relationship between communication and perceived value had the highest *β*-value of all the relationships. This finding is very relevant, since enhancing value thus turns into a strategy upon which the success of sports and health service organisations depends [41]. This has major ramifications, as value is a direct and indirect antecedent to both the satisfaction and future intentions of sports and health service users [21,34,35]. It is worth mentioning that girls had a significantly higher *β*-value than boys. This is in consonance with the study by Bernal-García [61], according to which, women attach greater importance to issues relating to the sacrifices (value) made in delivering the service. This finding may become a differentiating strategy in improving dropout rates in sports, which are higher among female adolescents [32].

Hypothesis 2, i.e., that communication is an antecedent of satisfaction, was corroborated in both boys and girls. Several studies [42,43] demonstrated that quality is an antecedent to satisfaction. In the present study, we went a step further by verifying that one dimension of quality is a direct antecedent to satisfaction. Recently, Álvarez-García et al. [62], in a similar study, were able to observe this relationship, but in an adult sample. For this reason, sports and health services that wish to increase the satisfaction levels of their users may use communication as a strategy to increase satisfaction levels even among the adolescent population.

In the literature on sports and health service management, studies have shown that service quality influences users’ future intentions [43,44]. In this study, a tool was used [50] that included three dimensions. This is why hypotheses 3, 4, and 5 were formulated in order to find out if communication is a predecessor to loyalty, adaptation to price, and responsiveness in users of these types of services. All three hypotheses were confirmed, so it is safe to say that sports organisations with adolescent users may use communication as a resource to improve adolescents’ future intentions.

Hypothesis 3 was confirmed, with male adolescents obtaining a higher *β*-value than female adolescents. Previous studies have found links between quality and loyalty in sports and health services [43,44] and even in similar services targeting adolescents [17].

Adaptation to price is another dimension included in the construct of future intentions. It was observed that communication is an antecedent to adaptation to price. This result confirms hypothesis 4. *β*-values were observed to be slightly higher in the group of boys. This result suggests that boys are more willing to pay more money for sports activities in educational institutions than girls. This result is interesting from an economic perspective, since most users of sports and health services are men, which could help to establish gender-differentiated pricing strategies. It should also be mentioned that girls are less willing to accept increases in the price of these activities, which, together with a higher dropout rate in sports, means that managers of sports and health services must handle this information carefully. They should try not to increase prices to prevent the percentage of girls who drop out from increasing even further.

Finally, the lowest *β*-values were found in the relationship between communication and responsiveness, with no differences in *β*-values between boys and girls. This finding confirms similar results in which responsiveness is the dimension with the lowest reported values. However, the effect of this dimension on loyalty has not been studied in depth, despite the existence of a significant and positive correlation between the two constructs [50]. An increasing number of studies examine the importance of word of mouth as one of the attitudinal behaviours of sports and health service users [61].

This result is important because adolescents who engage in physical activities organised by sports and health centres, in addition to perceiving a better state of health, have a healthier lifestyle, as they spend less time in “technological” activities and consume less alcohol and other substances harmful to their health [55]. It is also important that the relationship between communication and loyalty was confirmed, since an improvement in communication means that adolescents recommend the institution to their peers for their sports practice.

Although a major strength of this study is the fact that a large number of students of each sex participated. The noninclusion of sociodemographic variables is something we consider to be one of the principal limitations, especially in the case of socioeconomic status. This would have allowed us to better understand the profile of the adolescents and/or their families and therefore explain the differences in beta values between different dimensions.

As future lines of research, the sample of adolescents could be broadened to include those who only participate in noncompetitive extracurricular physical activities in order to check whether the results obtained are maintained or whether there are differences, given that, in our study, almost 91% took part in competitive activities. Furthermore, it would also be advisable to conduct a longitudinal study to check how the assessment of the service at these ages affects the adherence to sports in adolescents.

## 5. Conclusions

Organisational communication, one of the factors that serves as a measure of quality, is in itself an antecedent to the perceived value of and satisfaction with sports activities, programmes, and organisations in adolescents. Organisational communication is also an antecedent of future intentions, so it becomes a fundamental factor in getting adolescents to continue to engage in physical activity. The communication from sports and health services in educational institutions aimed at adolescents makes it possible to maintain and increase future intentions for physical/sports practice in this age group. It is necessary to mention that these activities are organised by educational institutions, which are responsible for the education of adolescents. Being aware of these relationships—between communication, value judgements, and future intentions—facilitates the implementation of interventions in aspects that the organisation of sports and health services may consider necessary. The improvement of the information provided and the contents, features, means, etc. available will increase the adolescents’ perceived value, satisfaction, and future intentions.

From a practical perspective, and with the aim of increasing the rates of participation in sporting activities among adolescents, avoiding them dropping out and improving their loyalty, educational institutions and sports centres must pay special attention to the communication factor. To this end, they must develop all sorts of strategies to increase communication, giving priority, for example, to the models, methods, and channels that are most effective and are most frequently used by adolescents. It is also very important that, in the strategies that are established, significant attention be paid to the aspects that are most relevant to girls, such as value or price, since they show a higher dropout rate at these ages.

Further research is needed on how different communication channels and diversified contents may increase physical/sports activity rates among adolescents and adults.

## Figures and Tables

**Table 1:** Physical/sports activities practised out-of-school hours.

	Sex		Total
	Male	Female	
Football	59.7%	5.7%	41.3%
Basketball	22.3%	49.0%	31.4%
Handball	10.4%	3.0%	7.9%
Dance	0.7%	27.5%	9.8%
Athletics	0.8%	2.5%	1.4%
Judo	2.0%	1.1%	1.7%
Gymnastics	0	2.5%	0.8%
Others	4.1%	8.7%	5.7%

**Table 2:** Descriptive statistics of the instrument.

Items	Mean	SD	Skewness	Kurtosis
The facilities have several means of receiving suggestions (suggestion box, bulletin board, website).	3.32	1.29	−0.286	−0.916
The information on the sports activities offered at the institution is appropriate.	3.74	1.00	−0.455	−0.261
The range of activities offered is constantly being updated.	3.60	1.11	−0.387	−0.514
It was a good decision to engage in extracurricular sports activities in this educational institution.	4.39	0.84	−1.334	1.354
I am glad that I enrolled in this educational institution.	4.38	0.85	−1.421	1.816
Joining this institution was a good decision.	4.28	0.91	−1.203	0.998
I am pleased to be enrolled in this institution.	4.29	0.96	−1.368	1.458
I am satisfied with the activity’s value for money.	3.72	1.05	−0.532	−0.101
I will share the positive aspects of this sports club with other people.	5.27	1.46	−0.721	0.264
I will recommend this sports centre to anyone seeking my advice.	5.36	1.42	−0.744	0.099
I will encourage my friends and family to participate in sports activities at this centre.	5.23	1.54	−0.722	−0.119
I would consider this club as my first choice for any sports service I might need.	5.21	1.45	−0.534	−0.257
In the next few years, I will take part in more sports activities in this club.	4.93	1.74	−0.514	−0.654
I will stay in this sports centre even if the prices are a little higher.	4.88	1.56	−0.466	−0.283
I am willing to pay a higher price than the prices charged at other gyms for the service I receive.	4.10	1.82	−0.068	−0.876
I will switch to another sports centre if I have any problems with the service here.	4.21	1.76	−0.107	−0.808
If I have a problem with the gym, I will complain to external entities such as the Spanish Organisation of Consumers and Users	3.79	1.88	0.047	−1.020
If I have a problem with the service, I will complain to the director of the sports centre.	4.49	1.844	−0.288	−0.856

**Table 3:** Descriptive statistics, internal consistency (diagonal), intercorrelations for study variables between extracurricular sports activities, Average Variance Extracted (AVE), and Composite Reliability (CR) (*N* = 1080).

Construct and Dimensions	M	SD	1	2	3	4	5	6	AVE	CR
1. Communication	3.55	0.93	(0.906)	0.434 **	0.360 **	0.461 **	0.304 **	0.119 **	0.676	0.862
2. Value	3.72	1.06			0.373 **	0.500 **	0.268 **	0.050	-	-
3. Satisfaction	4.33	0.77			(0.891)	0.562 **	0.267 **	0.037	0.754	0.925
4. Loyalty	5.20	1.16				(0.809)	0.495 **	0.118 **	0.583	0.873
5. Price	4.48	1.41					(0.534)	0.278 **	0.683	0.813
6. Responsiveness	4.16	1.45						(0.701)	0.627	0.834

Note. M = mean; SD = Standard Deviation; AVE = Average Variance Extracted; CR = Composite Reliability; ** *p* < 0.001.

**Table 4:** Adjustment statistics for the models, comparison between models using model 1 as correct.

Goodness-of-Fit Indices and Model Comparisons for Tested Models									Comparisons of Conditions Using Measurement Invariance Procedures			
Model	CMIN	DF	*p*	CMIN/DF	CFI	RMSEA	ECVI	AIC	ΔCMIN	ΔDF	ΔCFI	*p*
Model 0a	357.922	124	0.000	2.886	0.928	0.068	1.102	451.922				
Model 0b	201.385	124	0.000	1.624	0.922	0.061	1.769	295.385				
Model 1	766.453	248	0.000	3.091	0.910	0.052	1.228	954.453				
Model 2	809.900	261	0.000	3.103	0.905	0.052	1.251	971.900	43.447	13	0.005	0.000
Model 3	835.085	272	0.000	3.070	0.903	0.052	1.255	975.085	68.632	24	0.007	0.000
Model 4	838.428	273	0.000	3.071	0.902	0.052	1.257	976.428	71.975	25	0.008	0.000
Model 5	843.169	277	0.000	3.044	0.902	0.051	1.252	973.169	76.716	29	0.008	0.000
Model 6	892.621	295	0.000	3.026	0.897	0.051	1.270	986.621	126.168	47	0.013	0.000

Model 1 indicates no parameters constrained to be equal across groups; model 2, factor loadings constrained to be equal; model 3, observed variable intercepts and factor loadings constrained to be equal; model 4, residual variances, factor loadings, and observed variable intercepts constrained to be equal; model 5, factor variances and covariances, factor loadings, and observed variable intercepts constrained to be equal; model 6, factor means, factor loadings, observed variable intercepts, factor variances, and covariances constrained to be equal. Dif. CMIN = difference between model 1 and the other models; Dif. DF = difference between model 1 and the other models; *p* = significance level between models; *χ*2 (CMIN); degrees of freedom (DF); ratio between *χ*2 and the number of degrees of freedom (CMIN/DF); Comparative Fix Index (CFI); Root Mean Square Error of Approximation (RMSEA); Akaike Information Criterion (AIC); Expected Cross Validation Index (ECVI).

**Table 5:** Comparison between the standardised regression coefficients and hypothesis testing.

				Overall Sample			Boys			Girls		
			Hypothesis	*β*	*p*	Verification	*β*	*p*	Verification	*β*	*p*	Verification
Value	←	Comm.	1	0.496	0.000	Accepted	0.494	0.000	Accepted	0.525	0.000	Accepted
Satisfaction	←	Comm.	2	0.332	0.000	Accepted	0.336	0.000	Accepted	0.334	0.000	Accepted
Loyalty	←	Comm.	3	0.300	0.000	Accepted	0.316	0.000	Accepted	0.297	0.000	Accepted
Price	←	Comm.	4	0.271	0.000	Accepted	0.294	0.000	Accepted	0.282	0.000	Accepted
Response	←	Comm.	5	0.170	0.017	Accepted	0.156	0.012	Accepted	0.150	0.012	Accepted

## Abbreviations

EPOD2: Sports Organisations Perception Scale, version 2; AVE: Average Variance Extracted; SPSS: Statistical Package for the Social Sciences; CR: Composite Reliability; AMOS: Analysis of Moment Structure; CMIN: Chi-squared; DF: Degrees of Freedom; CFI: Comparative Fix Index; RMSEA: Root Mean Square Error of Approximation; AIC: Akaike Information Criterion; ECVI: Expected Cross-Validation Index.
